# Gastrointestinal mucormycosis due to *Rhizopus microsporus* following *Streptococcus pyogenes* toxic shock syndrome in an HIV patient: a case report

**DOI:** 10.1186/s12879-020-05548-9

**Published:** 2020-11-10

**Authors:** Thanaboon Yinadsawaphan, Popchai Ngamskulrungroj, Wipapat Chalermwai, Wutthiseth Dhitinanmuang, Nasikarn Angkasekwinai

**Affiliations:** 1 Department of Medicine, Faculty of Medicine Siriraj Hospital, Mahidol University, Bangkok, Thailand; 2 Department of Microbiology, Faculty of Medicine Siriraj Hospital, Mahidol University, Bangkok, Thailand; 3 Department of Pathology, Faculty of Medicine Siriraj Hospital, Mahidol University, Bangkok, Thailand

**Keywords:** Mucormycosis, Gastrointestinal mucormycosis, *Rhizopus microsporus*, Toxic shock syndrome, *Streptococcus pyogenes*, Bowel perforation

## Abstract

**Background:**

Gastrointestinal (GI) mucormycosis is a rare and often deadly form of mucormycosis. Delayed diagnosis can lead to an increased risk of death. Here, we report a case of GI mucormycosis following streptococcal toxic shock syndrome in a virologically suppressed HIV-infected patient.

**Case presentation:**

A 25-year-old Thai woman with a well-controlled HIV infection and Grave’s disease was admitted to a private hospital with a high-grade fever, vomiting, abdominal pain, and multiple episodes of mucous diarrhea for 3 days. On day 3 of that admission, the patient developed multiorgan failure and multiple hemorrhagic blebs were observed on all extremities. A diagnosis of streptococcal toxic shock was made before referral to Siriraj Hospital – Thailand’s largest national tertiary referral center. On day 10 of her admission at our center, she developed feeding intolerance and bloody diarrhea due to bowel ischemia and perforation. Bowel resection was performed, and histopathologic analysis of the resected bowel revealed acute suppurative transmural necrosis and vascular invasion with numerous broad irregular branching non-septate hyphae, both of which are consistent with GI mucormycosis. Peritoneal fluid fungal culture grew a grayish cottony colony of large non-septate hyphae and spherical sporangia containing ovoidal sporangiospores. A complete ITS1–5.8S-ITS2 region DNA sequence analysis revealed 100% homology with *Rhizopus microsporus* strains in GenBank (GenBank accession numbers KU729104 and AY803934). As a result, she was treated with liposomal amphotericin B. However and in spite of receiving appropriate treatment, our patient developed recurrent massive upper GI bleeding from Dieulafoy’s lesion and succumbed to her disease on day 33 of her admission.

**Conclusion:**

Diagnosis of gastrointestinal mucormycosis can be delayed due to a lack of well-established predisposing factors and non-specific presenting symptoms. Further studies in risk factors for abdominal mucormycosis are needed.

## Background

Mucormycosis is a rare and very serious opportunistic infection that is caused by fungi belonging to the order *Mucorales* (*Mucoromycotina*) [1], and it is associated with extremely high mortality [2]. This disease occurs most often in immunocompromised patients, such as those with poorly controlled diabetes mellitus, solid organ or hematopoietic stem cell transplantation, prolonged neutropenia, and those receiving deferoxamine chelation therapy or immunosuppressive therapy [3]. Six clinical forms of mucormycosis are generally recognized, including rhino-orbital, cerebral, pulmonary, cutaneous, disseminated, uncommon presentations, and one of the rarest manifestations - gastrointestinal involvement. All clinical manifestations of invasive mucormycosis share the same pathologic hallmark, which is angioinvasion and subsequent thrombosis leading to progressive tissue necrosis.

Increased morbidity and mortality is often caused by a delay in diagnosis and treatment since most patients present with non-specific gastrointestinal (GI) symptoms, and definite diagnosis can only be made by biopsy of the suspected area during surgery or endoscopy. Culturing gastric aspirates can also be used as an alternative method for diagnosis of GI mucormycosis, but its specificity is low due to possible contamination [2, 3]. Here, we present a case of gastrointestinal mucormycosis caused by *Rhizopus microspores* that developed shortly after the diagnosis of *Streptococcus pyogenes* toxic shock syndrome in a virologically suppressed HIV-infected patient. According to our review of the literature, this is only the second case of gastrointestinal mucormycosis following *S. pyogenes* toxic shock syndrome to be reported.

## Case presentation

A 25-year-old Thai woman with HIV infection and Grave’s disease was referred to Siriraj Hospital (Bangkok, Thailand) in February 2017 due to symptoms that included high-grade fever, vomiting, abdominal pain, and multiple episodes of mucousy diarrhea for 3 days. Our hospital is Thailand’s largest university-based national tertiary referral center.

She was diagnosed with HIV infection in 2004, and she maintained a virologically suppressed status via the use of tenofovir, emtricitabine, atazanavir, and boosted ritonavir with an absolute CD4^+^ count of 419 cells/mm^3^, and a CD4^+^ percentage of 31% at 5 months prior to this admission. Her Grave’s disease was diagnosed in 2005, it was successfully treated with radioactive iodine therapy, and it was maintained with oral thyroxine.

This patient was initially admitted to a private hospital where she presented with a hypotensive blood pressure of 65/27 mmHg and severe metabolic acidosis. She was diagnosed with severe gastroenteritis and septic shock, and she was empirically treated with ceftriaxone and metronidazole. However, during that admission, she developed multiorgan failure, including acute respiratory distress syndrome, acute kidney injury, and profound hypotension, even after receiving a high dose of both vasopressor and hydrocortisone. She was then intubated and antibiotic therapy was changed to meropenem. Before referral to our center on day 3 of her private hospital admission, multiple hemorrhagic blebs were observed on all extremities, and *Streptococcus pyogenes* was isolated from blood cultures. In contrast, conventional stool culture reported no growth.

Upon her arrival at Siriraj Hospital with a diagnosis of *S. pyogenes* toxic shock syndrome and multiorgan failure, she received supportive care with continuous renal replacement therapy. On day 5, her condition improved and vasopressor was discontinued. Antibiotic therapy was de-escalated from meropenem to penicillin and clindamycin. However, she still had persistent fever, bloody diarrhea, and hemorrhagic blebs at both hands. Necrotizing fasciitis at the left hand was suspected, but the patient and family refused amputation.

**Fig. 1:**
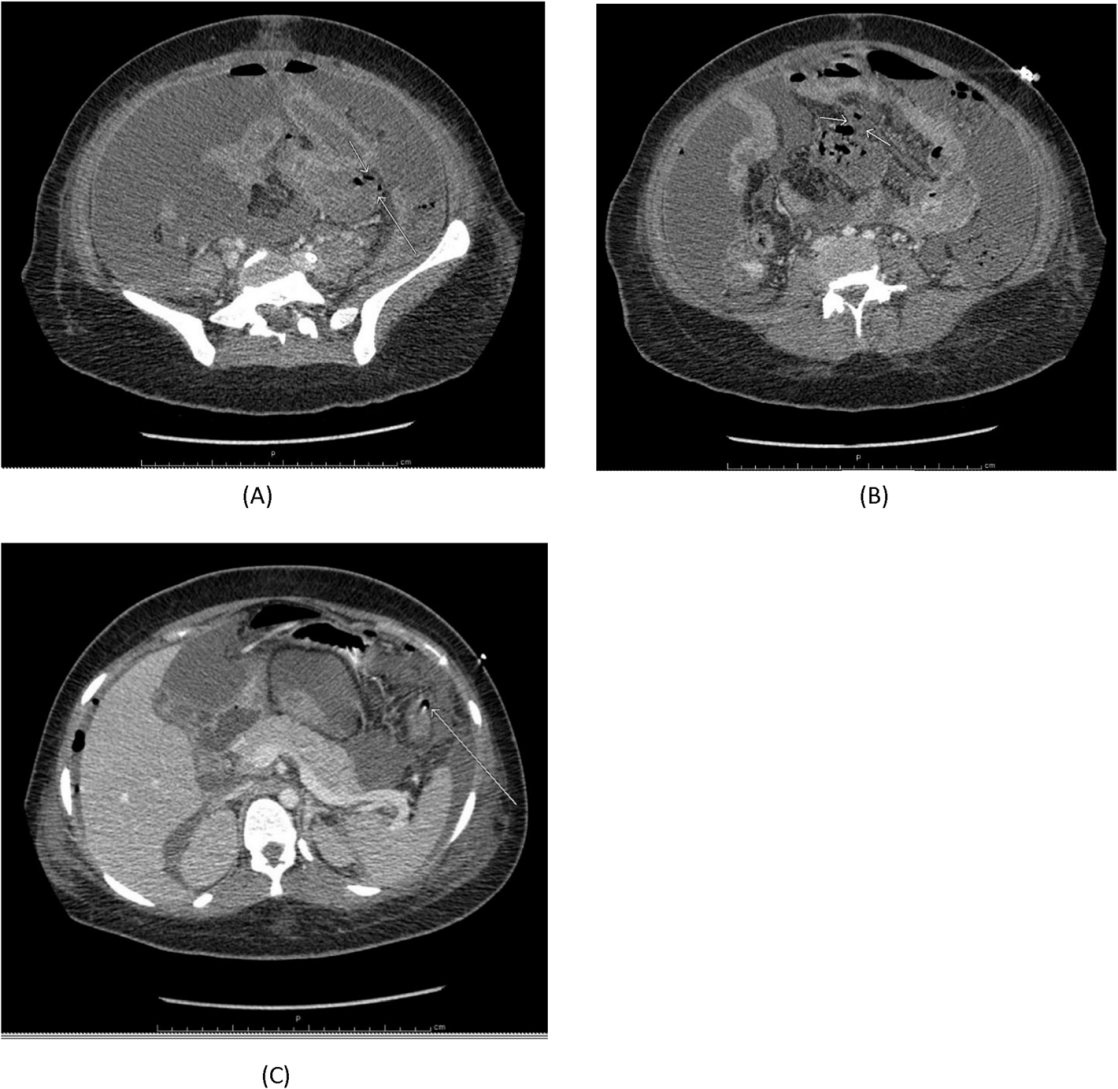
Contrast-enhanced computerized tomography (CT) scan of the abdomen revealed moderate pneumoperitoneum with intraperitoneal fluid. **a**, **b** Many sites of disrupted jejunal wall with mottled air from bowel lumen into nearby mesentery was observed, which is consistent with jejunal perforation. **c** Long segmental bowel wall thickening and marked enhancement along the jejunum, as well as bowel wall disruption at the splenic flexure of the colon, indicated the presence of air outside the colonic lumen

On day 10, she developed feeding intolerance with continued bloody diarrhea, distended abdomen with ascites, hypoactive bowel sounds, and high-grade fever with septic shock. The abdominal fluid analysis was consistent with secondary bacterial peritonitis. Abdominal computed tomography (CT) (Fig. 1) showed pneumoperitoneum with a perforation at the jejunum and splenic flexure. An emergency exploratory laparotomy was then performed, which revealed multiple ulcerative lesions and multiple necrotic foci at the jejunum and ileum. Segmental resection of the small bowel with abdominal toilet and temporary closure was performed. A tissue specimen from the resected bowel was sent for histopathologic analysis. On day 11, a second-look laparotomy was performed and new multiple small bowel necrotic foci were observed in addition to one perforation at the proximal ileum. In response, total small bowel resection and side-to-side jejunoileal anastomosis were performed.

Since initial ascitic fluid culture grew pan-drug resistant *Acinetobacter baumannii*, vancomycin-resistant *Enterococcus faecium* (VRE), carbapenem-resistant *K. pneumoniae* (CRE), and unspecified yeast species, her antimicrobial therapy was changed to piperacillin/tazobactam and fosfomycin, linezolid, colistin, and fluconazole.

**Fig. 2:**
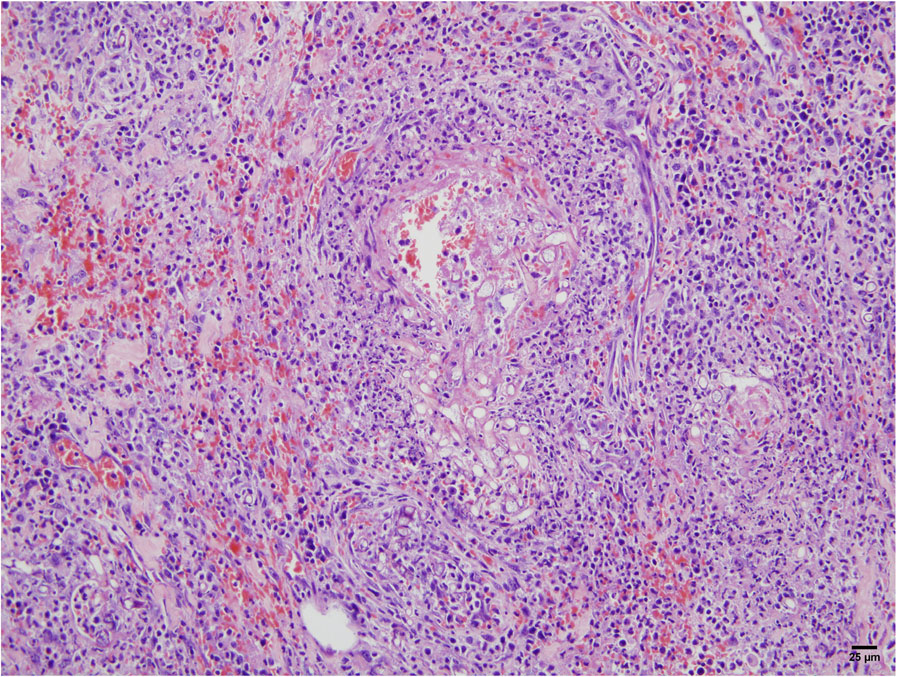
Vascular invasion by broad, irregular-shaped, non-septate hyphae with mycotic emboli surrounded by acute inflammation and necrosis that was identified in large areas of the small bowel by hematoxylin-eosin (H&E) staining (original magnification × 200)

**Fig. 3:**
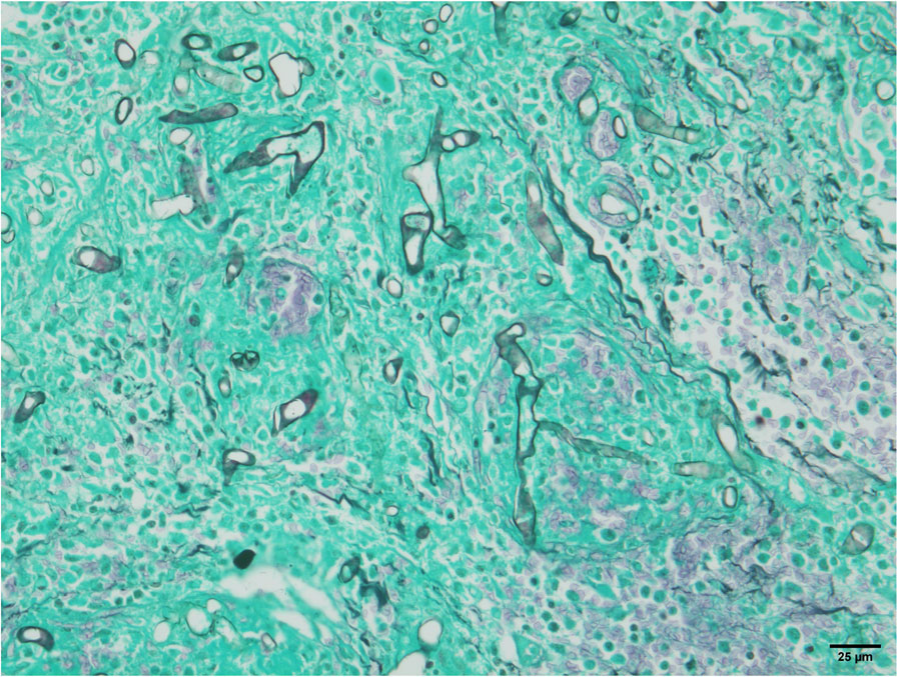
Morphology of fungal hyphae was visualized by modified Gomori methenamine-silver nitrate (GMS) stain (original magnification × 400)

Histopathology of the small bowel (Figs. 2 and 3) revealed acute suppurative and necrotizing granulomatous inflammation, transmural necrosis, and vascular invasion with numerous broad, irregular, branching non-septate hyphae, which is consistent with *Mucorales* fungi. Moreover, peritoneal fluid fungal cultures grew a grayish cottony colony on Sabouraud dextrose agar following 3 days of incubation at 25 °C, and demonstrated large non-septate hyphae and spherical sporangia containing ovoidal sporangiospores. To exclude other possible co-infection causing bowel necrosis, immunohistochemical stain for cytomegalovirus and an acid-fast stain for *Mycobacterium* were additionally performed on the bowel tissue biopsies, and all tests showed negative results. A complete ITS1–5.8S-ITS2 region DNA sequence analysis of the fungus showed 100% homology with *Rhizopus microsporus* strains in GenBank, including standard strains ATCC 22959 and ATCC 200758 (GenBank accession numbers KU729104 and AY803934). However, a variety level could not be determined due to a limitation of the ITS method [1].

Finally, the definite diagnosis of GI mucormycosis was made based on the evidence of intestinal tissue invasion by a non-septate fungus, which was later identified as *Rhizopus microsporus* by fungal culture and ITS sequence analysis. The patient was then treated as GI mucormycosis with liposomal amphotericin B at a dosage of 300 mg per day (5 mg/kg/day). After 12 days of amphotericin B treatment (day 33 of her admission), she developed recurrent massive upper GI bleeding from Dieulafoy’s lesion and she succumbed to her disease.

## Discussion and conclusion

Gastrointestinal (GI) mucormycosis is a rare disease with an extremely high mortality rate. It most commonly involves the stomach, followed by the colon and the small bowel [4], and transmission of the pathogen almost always occurs via the oral route [5]. The infection initially presents with non-specific symptoms, such as abdominal pain, vomiting, and abdominal distension. Patients typically develop gastrointestinal bleeding and fungating mass that can be observed on imaging. Angioinvasion is a potentially lethal complication because it can cause bowel necrosis that can lead to visceral perforation, peritonitis, and massive gastrointestinal bleeding [6, 7].

To our knowledge, this is only the second case of gastrointestinal mucormycosis after streptococcal toxic shock syndrome to be reported [8], and this is the first case report in Asia. Though it is possible that the patient had silent GI mucormycosis before streptococcal toxic shock syndrome, there is no evidence to prove such a hypothesis. According to the timeline of presentation, her condition improved after treatment for *Streptococcus pyogenes* bacteremia. Her abdominal symptom then worsened leading to a diagnosis of GI mucormycosis, which occurred 10 days after her diagnosis of streptococcal toxic shock syndrome. Similar to the first case report [8], there was no other known predisposing factor for mucormycosis, and the pathogen identified was also *Rhizopus microsporus*. Therefore, streptococcal toxic shock syndrome might be considered a newly discovered predisposing factor for this extremely rare form of mucormycosis. Increased risk of mucormycosis in this setting may be due to two possible mechanisms. First, since *S. pyogenes* produces a wide variety of exotoxins with superantigen activity [9], these circulating exotoxins may have resulted in cytokine-induced endothelial cell damage that facilitated and promoted fungal spore penetration into GI mucosa and blood vessels. Second, broad-spectrum antibiotic therapy given during *S. pyogenes* toxic shock syndrome adversely affects normal gut flora, especially anaerobic bacteria, and this can lead to significant overgrowth of pathogenic fungal organisms [10].

Interestingly, our patient developed GI mucormycosis without any well-established predisposing factor. Her absolute CD4^+^count of 419 cells/μL was not diminished to a level that rendered her susceptible to opportunistic pathogens (< 200 cells/μL). In fact, among 297 patients with invasive fungal infection in a large retrospective study of 1630 autopsies of patients with acquired immunodeficiency syndrome (AIDS) during 1984 to 2002 [11], only two patients were diagnosed mucormycosis with pulmonary and CNS involvement. Additionally, a systematic review of mucormycosis in 67 HIV-infected patients revealed a very low CD4^+^ count (median: 47 cells/μL, interquartile range: 17–100 cells/μL) and one or more predisposing factors [12].

Early diagnosis and treatment are crucial to prevent complications and death. However, early diagnosis is difficult due to non-specific sign(s) and symptom(s), and the lack of a diagnostic tool to confirm the disease. Although circulating *Mucorales* DNA detection is a new and promising diagnostic tool, it is not yet widely used in routine diagnostic testing [13]. In the present case, tissue specimens from the site of infection were needed for culture or histopathology to confirm the diagnosis. Moreover and unfortunately, a definite diagnosis of our patient’s condition was not made until much later in the clinical course of her disease.

The mainstay of treatment for mucormycosis is the surgical debridement of necrotic tissue to prevent the progression of the infection to vital structures or dissemination to other sites. Reduced blood supply to tissue due to vessel thrombosis means that adequate surgical debridement of these compromised tissues can enhance the penetration of antifungal agents into still viable infected tissue. Additionally, antifungal therapy should be initiated when mucormycosis is highly suspected [14, 15]. Polyene antifungal agent, particularly lipid formulation of amphotericin B (LAmB), is preferred to amphotericin B deoxycholate (AmB) because of its comparatively lower level of nephrotoxicity when given at higher doses. Among extended-spectrum triazole antifungal agents, isavuconazole and posaconazole demonstrated activity against *Mucorales* in vitro [15, 16]. Posaconazole monotherapy was shown to be inferior to AmB for the treatment of mucormycosis in mice [17]. However, isavuconazole showed activity against mucormycosis with efficacy similar to AmB in the VITAL study [18]. Combination therapy with LAmB and caspofungin was reported to yield comparatively better treatment outcomes in patients with rhino-orbital-cerebral mucormycosis [19]. Despite the extensive surgical removal of infected tissue and LAmB treatment in our patient, death was a near inevitability since the infection was too advanced to be controlled. This emphasizes the importance of early diagnosis to achieve a better treatment outcome.

In conclusion, GI mucormycosis remains a diagnostic and therapeutic challenge. Nonspecific complaints and the lack of well-established predisposing factors in our patient delayed the diagnosis of GI mucormycosis, and this resulted in worse outcome and death – even after appropriate treatment was given. Studies in risk factors for abdominal mucormycosis are needed.

## Data Availability

All data generated or analysed during this study are included in this published article.

## Abbreviations

GI: Gastrointestinal
CT: Computed tomography
VRE: Vancomycin-resistant *Enterococcus faecium*
CRE: Carbapenem-resistant *Enterobacteriaceae*
LAmB: Lipid formulation of amphotericin B
